# Supplementary material for: Novel introductions of human-origin H3N2 influenza viruses in swine, Chile
Source: Front Vet Sci. 2025 Jan 9;11:1505497. doi: 10.3389/fvets.2024.1505497 (PMC11755890; doi:10.3389/fvets.2024.1505497)

Supplementary Table 1.- Description of the total samples by type and their percentage of positivity to RT-PCR IAV.

Sample	Total	RT-PCR IAV+	Positivity
BS	64	23	35,9%
L	26	2	7,7%
NS	1070	90	8,4%
OF	32	12	37,5%
TS	3	0	0,0%
**Total**	**1195**	**127**	**10,6%**

Supplementary Table 2.- Description of the closest sequences detected with BLASTn for each genome segment of the A/swine/O'Higgins/VN1401-7442/2023 and A/swine/O'Higgins/VN1401-7826/2024 viruses.

**Sequence BLASTn**	**A/swine/O'Higgins/VN1401-7442/2023**				**A/swine/O'Higgins/VN1401-7826/2024**			
**Segment**	**Strain name**	**Accesion number**	**Host**	**Percent identity**	**Strain name**	**Accesion number**	**Host**	**Percent identity**
**HA**	A/Human/New York City/PV63055/2022(H3N2)) segment 4 hemagglutinin (HA) gene, complete cds.	OP432929.1	Human	99.26%	(A/Human/New York City/PV63324/2022(H3N2)) segment 4 hemagglutinin (HA) gene, complete cds.	OQ068146	Human	98.98%
**NA**	Influenza A virus genome assembly, segment: 6.	OY283278	Human	99.66%	A/swine/Ohiggins/VN1401-5105/2020(HxN2)) segment 6 neuraminidase (NA) gene, complete cds.	MZ005159	Swine	97.20%
**PB2**	Influenza A virus (A/Human/New York City/PV63055/2022(H3N2)) segment 1 polymerase PB2 (PB2) gene, complete cds	OP432930.1	Human	99.83%	Influenza A virus (A/swine/Ohiggins/VN1401-5016/2020(H1N2)) segment 1 polymerase PB2 (PB2) gene, complete cds	MZ005153.1	Swine	97.52%
**PB1**	A/Human/New York City/PV63357/2022(H3N2)) segment 2 polymerase PB1 (PB1) and PB1-F2 protein (PB1-F2) genes, complete cds.	OQ059059	Human	99.91%	A/swine/Ohiggins/VN1401-5108/2020(H1N2)) segment 2 polymerase PB1 (PB1) gene, complete cds; and nonfunctional PB1-F2 protein (PB1-F2) gene, complete sequence.	MZ013916	Swine	98.12%
**PA**	Influenza A virus (A/Human/New York City/PV82899/2022(H3N2)) segment 3 polymerase PA	OQ787306.1	Human	99.69%	Influenza A virus (A/swine/Ohiggins/VN1401-5101/2020(H1N1)) segment 3 polymerase PA (PA) and	MZ005177	Swine	97.94%

	(PA) and PA-X protein (PA-X) genes, complete cds				PA-X protein (PA-X) genes, complete cds.			
**NP**	Influenza A virus (A/Human/New York City/PV63367/2022(H3N2)) segment 5 nucleocapsid protein (NP) gene, complete cds	OQ059172.1	Human	99.55%	A/swine/Ohiggins/VN1401-5092/2020(HxN2)) segment 5 nucleocapsid protein (NP) gene, complete cds.	MZ005183	Swine	98.34%
**M**	Influenza A virus genome assembly, segment: 7.	OX411443.1	Human	99.90%	Influenza A virus (A/swine/Ohiggins/VN1401-5108/2020(H1N2)) segment 7 matrix protein 2 (M2) and matrix protein 1 (M1) genes, complete cds	MZ005134.1	Swine	98.83%
**NS**	A/WA/31872/2022(H3N2)) segment 8 nuclear export protein (NEP) and nonstructural protein 1 (NS1) genes, complete cds.	OQ180177.1	Human	99.66%	Influenza A virus (A/swine/Rancagua/VN1401-2807/2017(H1N2)) segment 8 nuclear export protein (NEP) and nonstructural protein 1 (NS1) genes, complete cds	MH346870.1	Swine	98.54%

Supplementary Figure 1. PB2 phylogenetic tree. The sequences are color-coded for clarity: red Chilean swine, green Chilean human, blue USA swine, and black indicates humans from the USA. For clarity, the isolates A/swine/O’Higgins/VN1401-7826/2024 (H3N2) and A/swine/O’Higgins/VN1401-7442/2024 (H3N2) are depicted with a red circle.

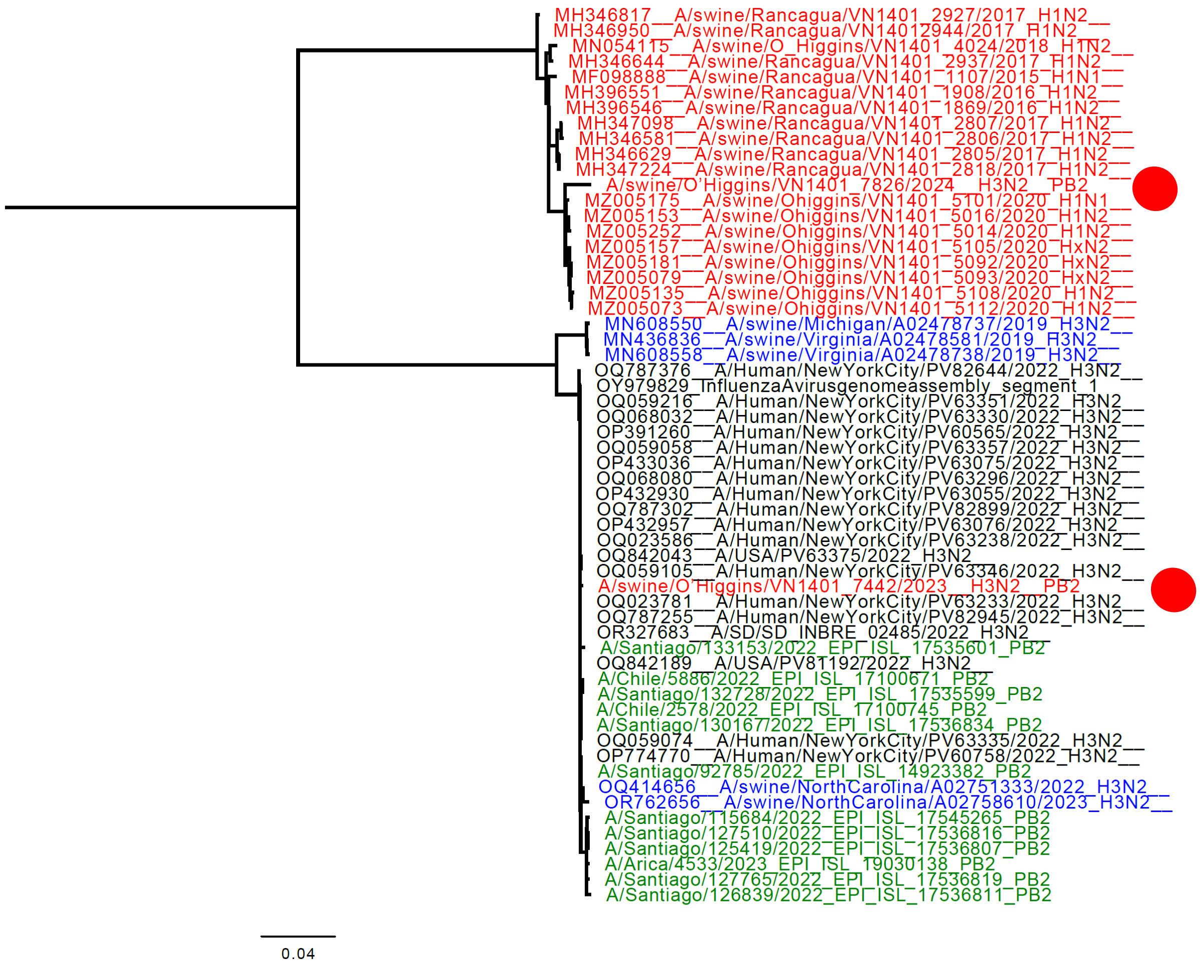

Supplementary Figure 2. PB1 phylogenetic tree. The sequences are color-coded for clarity: red Chilean swine, green Chilean human, blue USA swine, and black indicates humans from the USA. For clarity, the isolates A/swine/O'Higgins/VN1401-7826/2024 (H3N2) and A/swine/O'Higgins/VN1401-7442/2024 (H3N2) are depicted with a red circle.

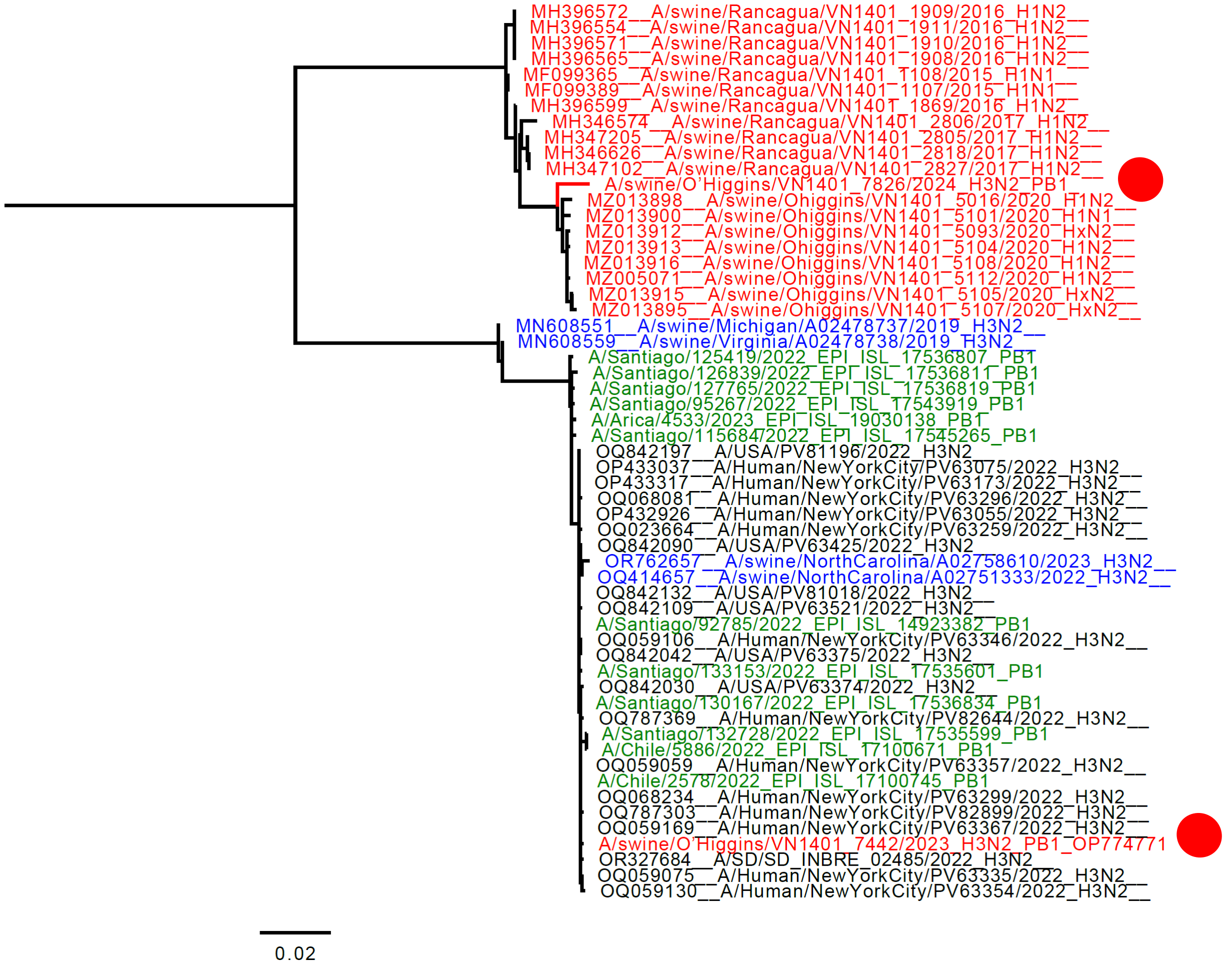

Supplementary Figure 3. PA phylogenetic tree. The sequences are color-coded for clarity: red Chilean swine, green Chilean human, blue USA swine, and black indicates humans from the USA. For clarity, the isolates A/swine/O'Higgins/VN1401-7826/2024 (H3N2) and A/swine/O'Higgins/VN1401-7442/2024 (H3N2) are depicted with a red circle.

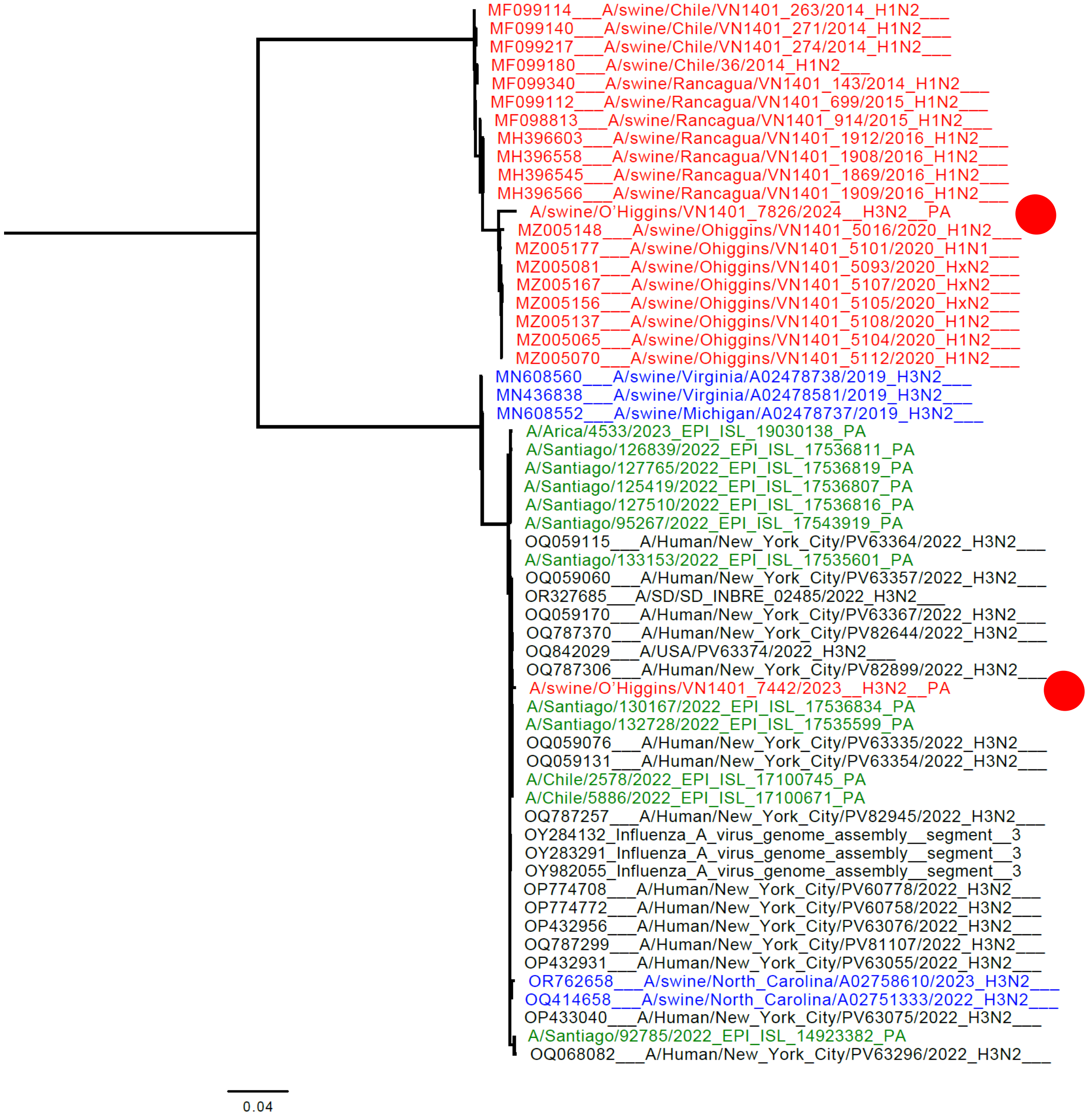

Supplementary Figure 4. NP phylogenetic tree. The sequences are color-coded for clarity: red Chilean swine, green Chilean human, blue USA swine, and black indicates humans from the USA. For clarity, the isolates A/swine/O'Higgins/VN1401-7826/2024 (H3N2) and A/swine/O'Higgins/VN1401-7442/2024 (H3N2) are depicted with a red circle.

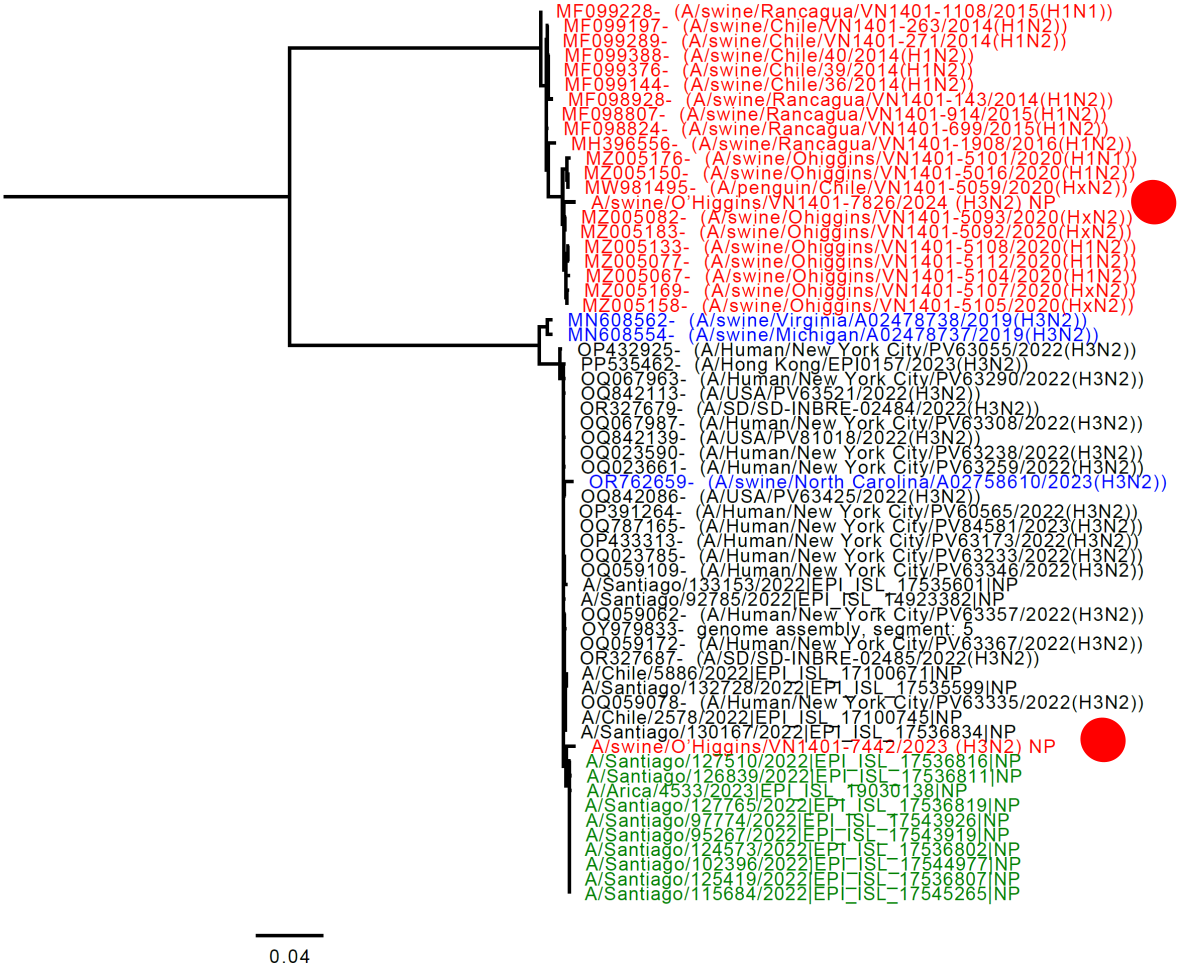

Supplementary Figure 5. M phylogenetic tree. The sequences are color-coded for clarity: red Chilean swine, green Chilean human, blue USA swine, and black indicates humans from the USA. For clarity, the isolates A/swine/O'Higgins/VN1401-7826/2024 (H3N2) and A/swine/O'Higgins/VN1401-7442/2024 (H3N2) are depicted with a red circle.

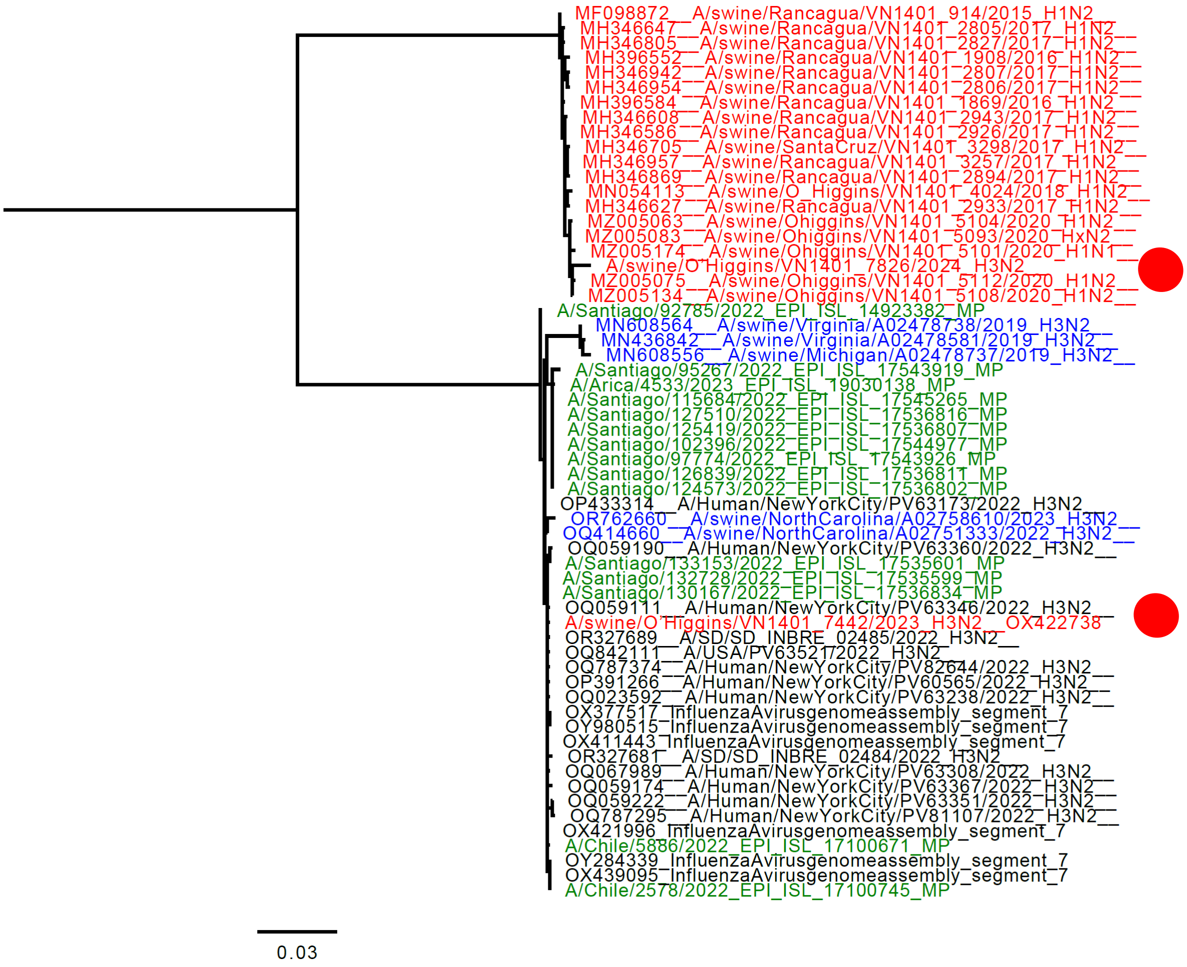

Supplementary Figure 6. NS phylogenetic tree. The sequences are color-coded for clarity: red Chilean swine, green Chilean human, blue USA swine, and black indicates humans from the USA. For clarity, the isolates A/swine/O'Higgins/VN1401-7826/2024 (H3N2) and A/swine/O'Higgins/VN1401-7442/2024 (H3N2) are depicted with a red circle.

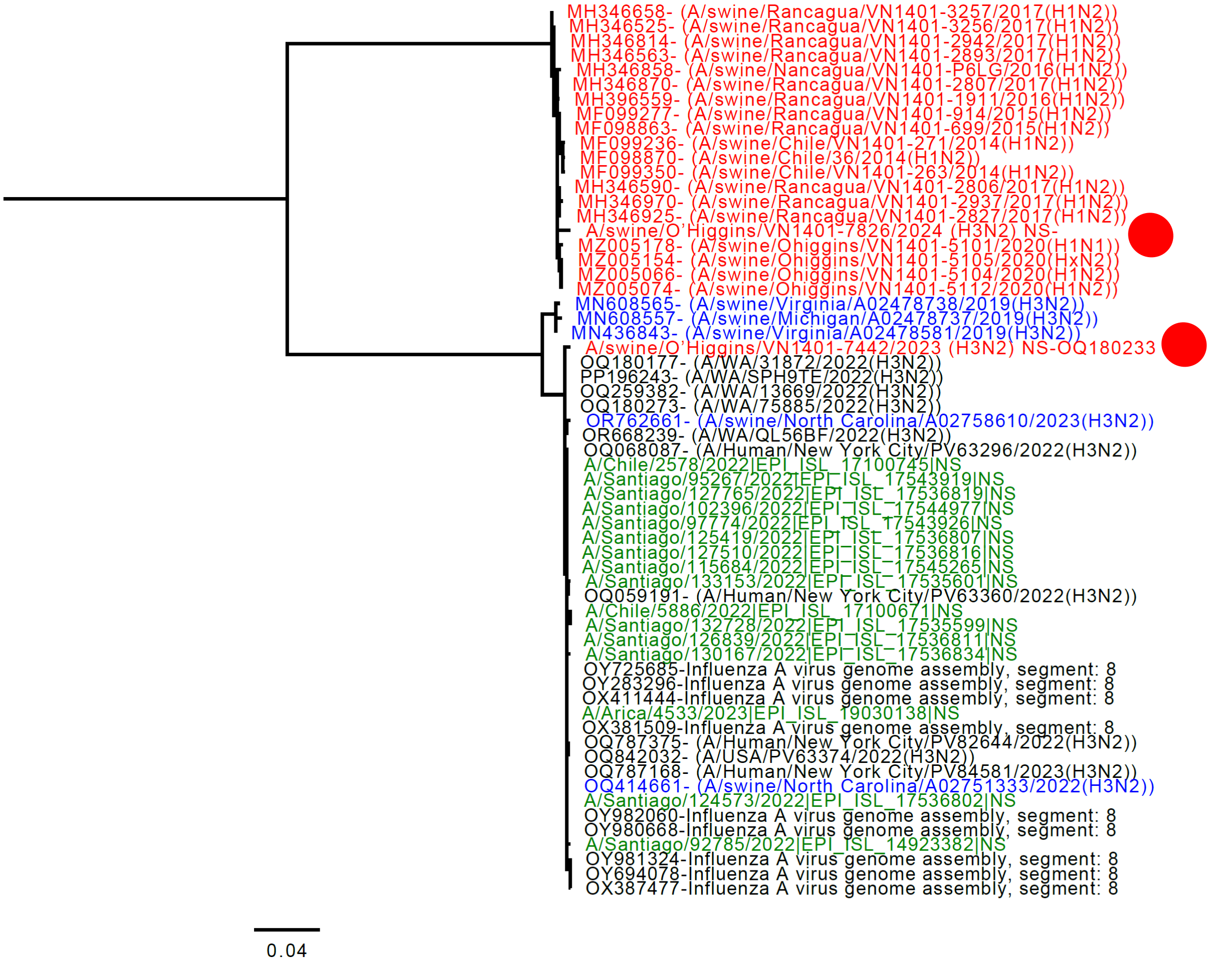

Supplementary Figure 7. Dual Brothers recombination analysis suggest that A/swine/O'Higgins/VN1401-7826/2024 (H3N2) is a reassortant strain in the HA gene. The topologies show the probabilities of the three most likely phylogenetic trees at each site along the alignment, reflecting the closest related sequences to the potential reassortant strain. Specifically, A/swine/O'Higgins/VN1401-5112/2020 (H1N2) (red line) is highly similar to the reassortant strain across the entire genome (100%) except for the HA segment (0%). In contrast, A/Human/New York City/PV63324/2022 (H3N2) shares similarity with the reassortant strain only in the HA segment (100%).

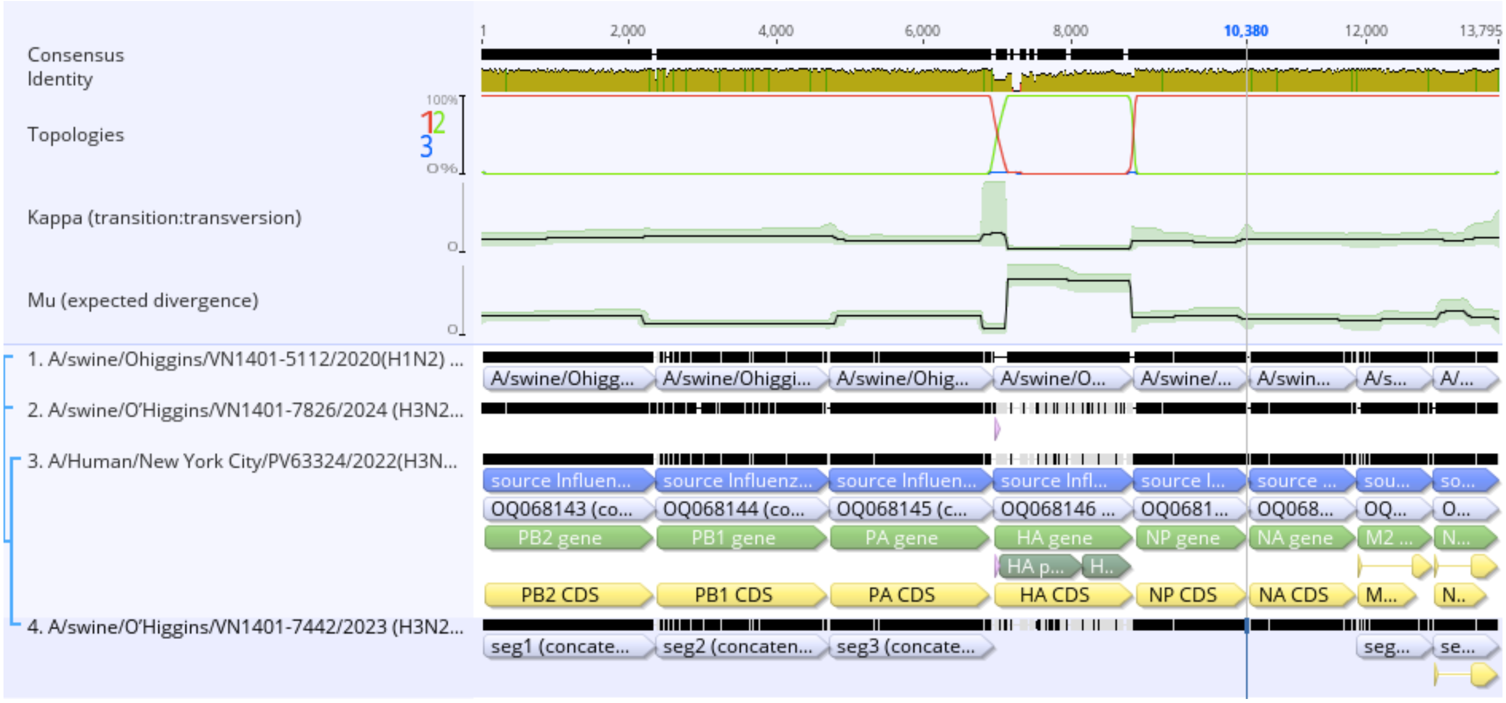

Supplementary Figure 8. RDP4 v.4.101 overall results. Recombination analysis suggests that A/swine/O'Higgins/VN1401-7826/2024 (H3N2) is a reassortant strain

RECOMBINATION EVENT NUMBER 1
Beginning breakpoint: 7015 in alignment (6986 without gaps)
Beginning breakpoint 99% CI: 6887 - 7124 in alignment (6861 - 7095 without gaps)
Ending breakpoint: 8836 in alignment (8665 without gaps)
Ending breakpoint 99% CI: 8800 - 8873 in alignment (8629 - 8701 without gaps)
Recombinant: A/swine/Oâ€™Higgins/VN1401-7826/2024_(H3N2)_concatenated
Major Parent: A/swine/Ohiggins/VN1401-5112/2020(H1N2)_concatenated Concatenation of 8 sequences (98.6% similarity)
Minor Parent: A/Human/New_York_City/PV63324/2022(H3N2)_concatenated Concatenation of 8 sequences (99.2% similarity)

Confirmation Table

Methods	# seqs detected in	Av. P-Val
RDP	1	4E-30 X 10^{-300}
GENECONV	1	3,064 X 10^{-307}
BootScan	--	--
MaxChi	1	3,832 X 10^{-70}
Chimaera	1	1,781 X 10^{-70}
SiScan	1	4,964 X 10^{-86}
3Seq	1	4E-30 X 10^{-300}
LARD	--	--
Phylpro	--	--

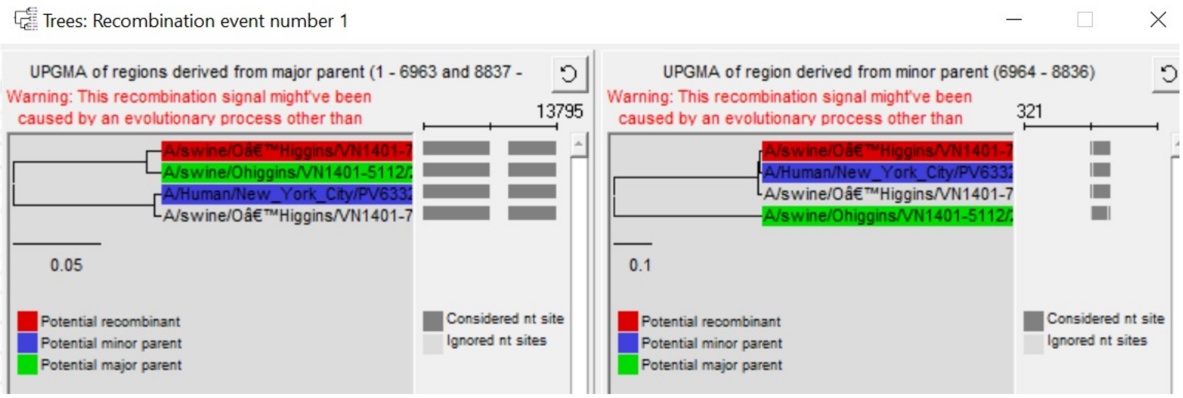

Supplement: Supplementary file 1 [file Data_Sheet_1.PDF]
